# Hybrid C-V and Star Flap Technique for Nipple Reconstruction: A Case Report

**DOI:** 10.7759/cureus.111286

**Published:** 2026-06-22

**Authors:** Tejaswini Malapati, Manal M Khan, Gaurav Chaturvedi, Abhinav Singh, Devesha Rathour

**Affiliations:** 1 Plastic and Reconstructive Surgery, All India Institute of Medical Sciences, Bhopal, Bhopal, IND

**Keywords:** c-v flap, de-epithelialized dermal component, hybrid technique, nipple–areola complex reconstruction, star flap

## Abstract

Nipple-areola complex (NAC) reconstruction is the final step in breast reconstruction and significantly influences aesthetic outcome and patient satisfaction. Progressive loss of nipple projection remains a persistent challenge despite numerous local flap techniques. We present a hybrid technique combining C-V and star flaps to enhance central bulk, support projection, and preserve vascularity. A 28-year-old woman underwent bilateral NAC reconstruction three months after implant-based breast reconstruction for hypoplasia. The reconstructed nipples healed without flap necrosis or other complications, with preserved symmetry, and the patient was satisfied with the outcome; some reduction in projection was noted over the follow-up period, although projection was not objectively measured. By integrating the volumetric advantage of the star flap with the vascular reliability of the C-V flap and adding a de-epithelialized triangular dermal component, this hybrid design was technically feasible and produced a satisfactory short-term result. As with established techniques, early projection loss may occur; whether the design offers any advantage in projection retention cannot be determined from a single short-term case and requires comparative study with objective measurement and longer follow-up.

## Introduction

Reconstruction of the nipple-areola complex (NAC) is widely regarded as the final stage of breast reconstruction [1]. Despite technical refinements, the most common drawback remains progressive flattening of the reconstructed nipple, with reported projection loss of 19% at one year [2,3]. The C-V flap, popularised by Little [4], remains the workhorse flap due to its simplicity and vascular reliability, yet often has modest projection. Briefly, the C-V flap raises a central C flap and two lateral V limbs that are wrapped to form the neo-nipple, while the star flap uses three converging triangular limbs that are elevated and folded to create projection. Local flap techniques include the star flap, introduced by Anton et al. [5], which is the most commonly utilised and achieves greater initial projection but may show progressive reduction or tip ischemia in scarred fields. Losken et al. [6] reported a long-term evaluation of the C-V flap, confirming its vascular reliability but modest projection. To overcome these shortcomings, various modifications have been proposed [7], including changes in design and the use of acellular dermal matrix or other augmentation grafts [8,9]. Techniques incorporating de-epithelialized dermal components within star flap reconstruction have previously been reported [10]. However, these approaches focus on modifications of the star flap alone. We have been using the star flap in our practice but have noticed a gradual decrease in nipple projection and a loss of nipple bulk over time. To overcome these limitations, we devised a hybrid C-V + star flap technique that uses a de-epithelialized dermal flap for nipple reconstruction. Unlike previously described star flap modifications incorporating dermal reinforcement (e.g., Lee et al. [10]), our technique combines elements of both the CV and star flaps within a single hybrid design, adding a de-epithelialized dermal core to a C-V framework rather than altering star flap geometry alone. This report details the surgical and operative designs and outcomes.

## Case presentation

**Figure 1:**
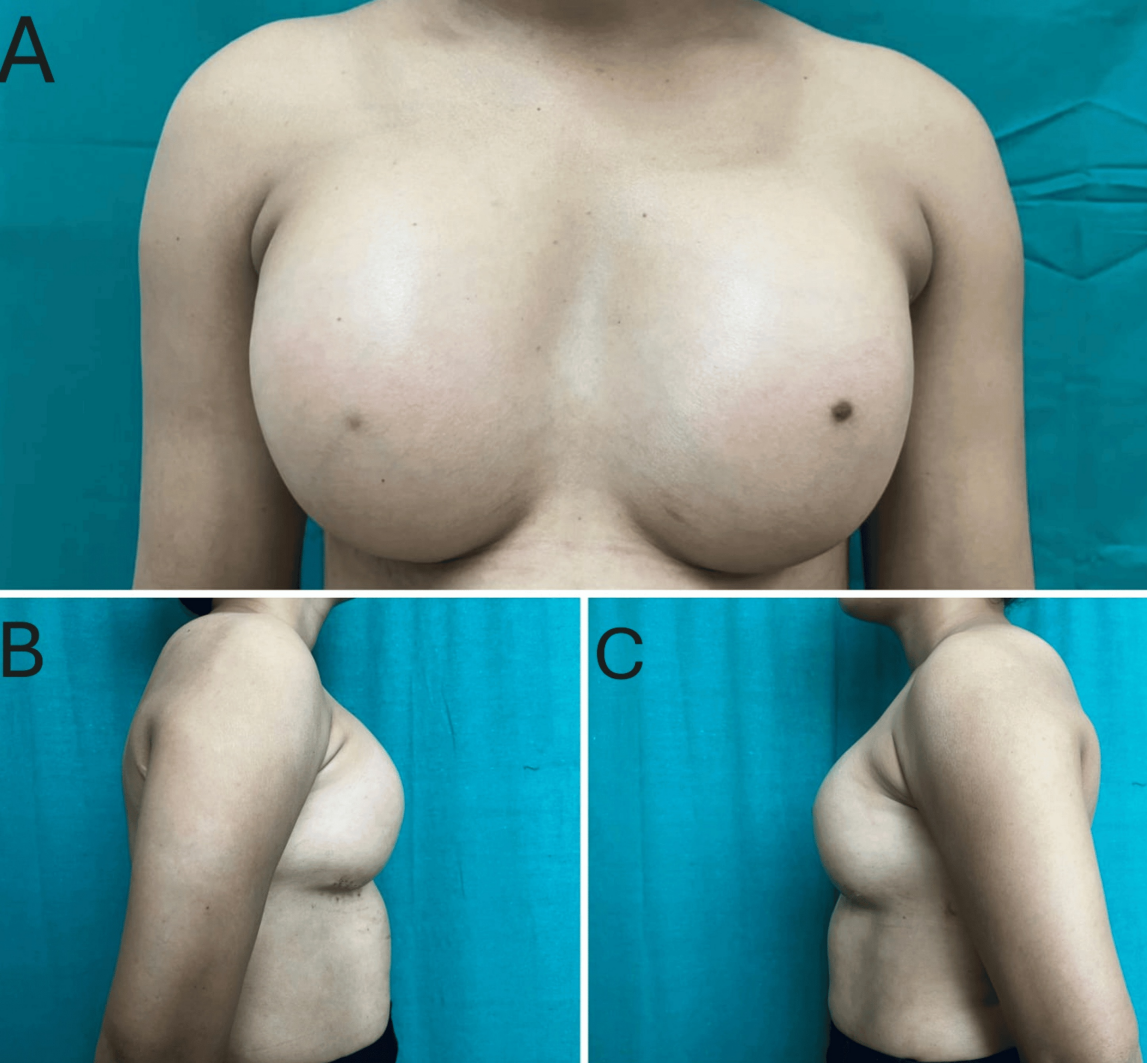
Preoperative photographs A: Frontal view, B: right lateral view, and C: left lateral view demonstrating breast projection

A 28-year-old woman with bilateral breast hypoplasia previously underwent implant-based bilateral breast reconstruction using round, semismooth implants (265 mL each; base diameter of 101 mm, projection of 49 mm) placed in the dual-plane position. She was a non-smoker, with a body mass index of 21.6 kg/m², no significant past medical or surgical history, and no prior radiotherapy. Three months postoperatively, she presented for NAC reconstruction. Her postoperative course following breast reconstruction was uncomplicated. Clinical examination revealed both breasts had satisfactory volume and contour, with well-healed inframammary scars (Figures 1A-1C). The implants were soft, and there was no evidence of capsular contracture or wound-related complications.

**Figure 2:**
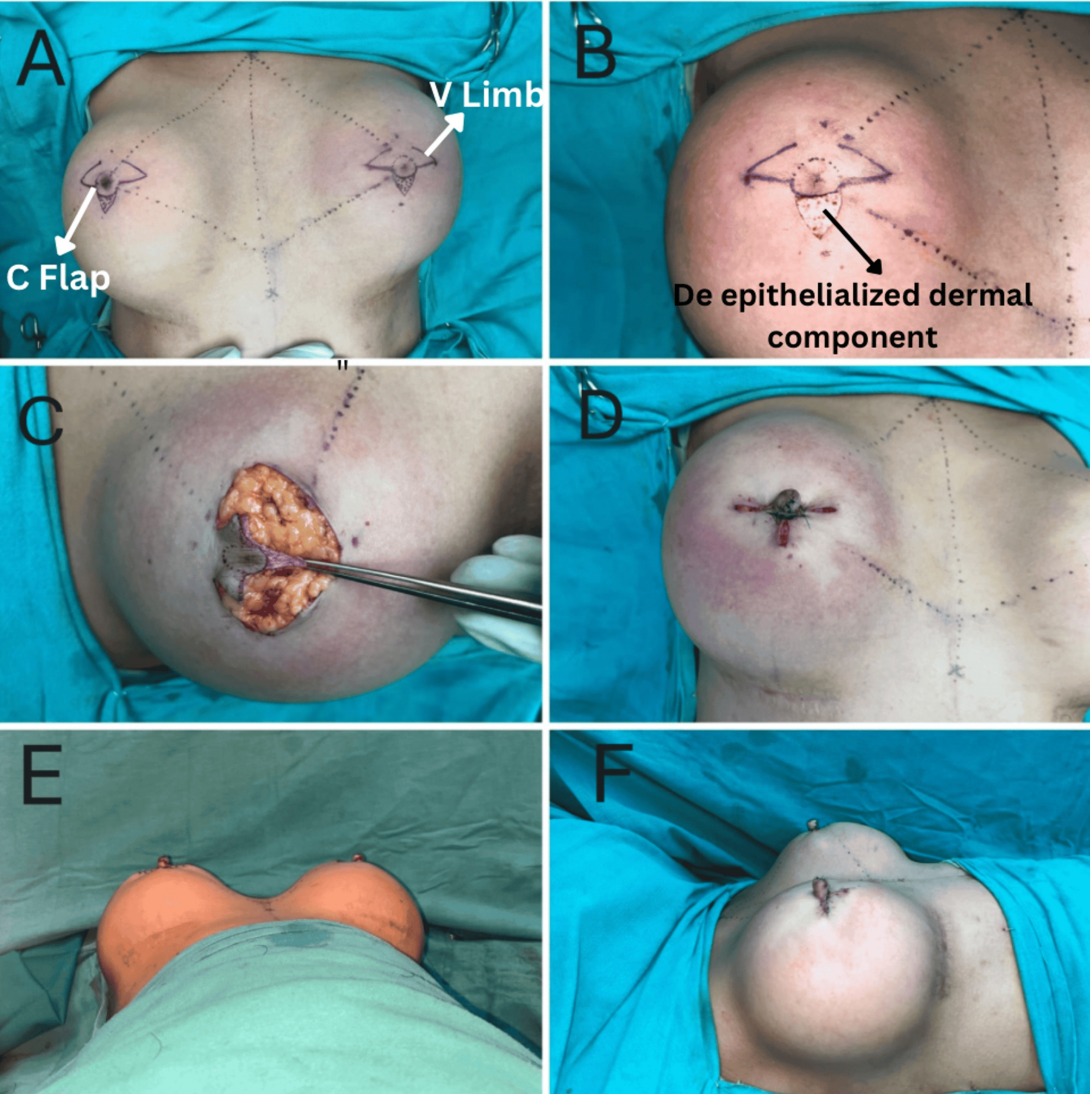
Intraoperative photographs A: On-table markings showing planned nipple positions and outlines of the hybrid C-V and star flap design. B: De-epithelialized triangular dermal component. C: Elevation of the hybrid flap with the de-epithelialized triangular dermal component. D: Immediate intraoperative inset. E-F: Foot end view and oblique view demonstrating bilateral nipple projection

Preoperative planning was conducted with the patient standing. Symmetric nipple positions were marked bilaterally using fixed anatomical landmarks, including the midline, sternal notch, inframammary folds, and mid-clavicular lines (Figure 2A).

The procedure was performed as an inpatient procedure under general anaesthesia, with local infiltration of 2% lignocaine with 1:200,000 adrenaline at the flap sites. On each breast, a hybrid flap design was marked, consisting of two lateral V limbs and a central C flap. The central C flap was extended distally into an additional V-shaped triangular segment, which was de-epithelialized to create a dermal flap (Figure 2B). Care was taken to preserve the base of the C flap to maintain vascularity. The dimensions of the C flap determined the diameter of the reconstructed nipple, while the width of the lateral V limbs determined nipple projection. The C flap measured approximately 1-1.2 cm in diameter, the lateral V limbs approximately 1.0 cm in width and length, and the de-epithelialized triangular dermal segment approximately 1×1 cm at its base.

Following incision, the V limbs were elevated in the subcutaneous plane and wrapped around the central C flap. The de-epithelialized triangular dermal segment was rolled into the core of the neo-nipple to enhance bulk (Figures 2C-2D). The flaps were sutured using 4-0 Monocryl, and the donor sites were closed with interrupted 4-0 nylon sutures. Immediate intraoperative inset demonstrated good projection (Figures 2E-2F). Topical antibiotic ointment was applied, followed by a waterproof dressing with a central window to allow for flap monitoring.

All flaps demonstrated good perfusion in the immediate postoperative period. The first dressing change was done on postoperative day two, followed by alternate-day dressings for two weeks. Sutures were removed on postoperative day 14. The postoperative period was uneventful, with no evidence of flap necrosis, wound dehiscence, or infection, and no significant contour irregularity or colour mismatch was noted at follow-up.

**Figure 3:**
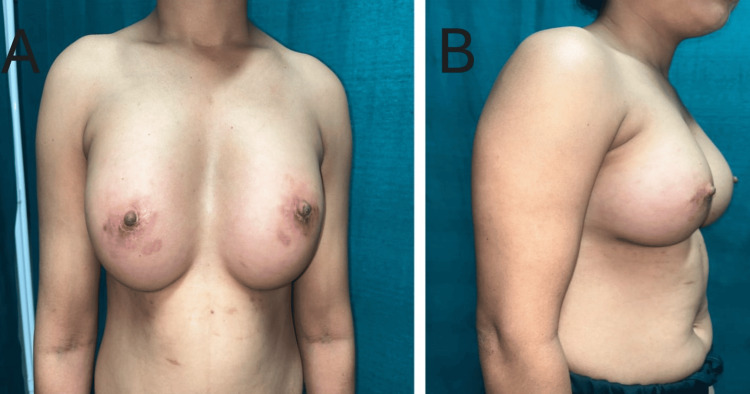
Postoperative photographs at three-month follow-up A: Frontal view demonstrating symmetric nipple position and contour. B: Lateral view showing maintained nipple projection

At the three-month follow-up, both reconstructed nipples retained symmetry, although some reduction in projection was noted compared with the immediate postoperative result. Projection was not formally measured (Figures 3A-3B).

Areola reconstruction by tattooing was advised following complete healing of the reconstructed nipples. The patient expressed high satisfaction with the outcome. 

Written informed consent was obtained from the patient for publication of clinical details and photographs.

## Discussion

Loss of long-term projection remains the principal limitation of nipple reconstruction. The C-V flap provides dependable vascularity but often limited height, while the star flap offers superior initial projection with potential late flattening. Structural augmentation techniques have aimed to address this issue. The skate flap is another widely used alternative, providing reliable initial projection through a different flap geometry, though it similarly demonstrates progressive projection loss over time [3]. Augmentation strategies using autologous, allogeneic, or synthetic grafts, including acellular dermal matrix, can reduce projection loss but may increase the risk of flap-related complications [8,9]. Lee et al. reported a star flap modification incorporating trapezoidal wings with de-epithelialized dermal reinforcement to enhance nipple projection [10]. Our approach also uses dermal support, but the design differs in that the dermal component is incorporated within a hybrid C-V and star configuration rather than within a modified star flap alone. Our hybrid technique integrates the volumetric advantage of the star flap with the vascular reliability of the C-V flap. It adds a de-epithelialized dermal component to increase central bulk. In this case, some early reduction in nipple projection was observed clinically over the three months, consistent with the well-recognised pattern of early flattening [2,3]; projection was not objectively quantified. Whether the hybrid design confers any advantage in projection retention cannot be established from a single, unmeasured, short-term case and would require prospective comparative evaluation against standard C-V or star flap reconstruction, with objective measurement at twelve months or beyond. Wrapping V limbs around a robust central column is intended to redistribute tension and support projection; structural reinforcement has been proposed as a means of achieving more predictable projection [11], although this could not be assessed objectively in the present short-term case.

The operative steps are straightforward and do not add significant complexity to standard nipple reconstruction. For surgeons already familiar with local flap techniques, the modification is simple to reproduce and can be incorporated into routine practice without difficulty. Donor-site morbidity is minimal, as the additional de-epithelialized segment is harvested from within the existing flap design without extending the incision. The technique added little operative time compared with a standard C-V or star flap and required no additional equipment, supporting its practical adoption.

This report has some important limitations. It describes a single case from a single surgeon at a single institution, which precludes generalisability. Projection was not objectively measured, limiting quantitative assessment of the outcome. The three-month follow-up is short relative to the known timeline of projection loss, which has been reported to occur progressively over 12 months [2,3]; long-term (≥12-month) follow-up with objective measurement would be required to determine whether this hybrid technique improves projection maintenance compared with established methods. There was no comparator technique, and patient satisfaction was recorded as an informal observation rather than with a validated patient-reported outcome measure. Prospective comparative studies are needed.

## Conclusions

This hybrid modification combining the C-V and star flaps with a de-epithelialized dermal core was technically feasible and produced a satisfactory short-term result in a single case, without flap necrosis or other complications. Projection was not objectively measured, and some early reduction was observed clinically. Larger prospective comparative studies with objective projection measurement and follow-up of at least 12 months are required to determine whether this design offers any advantage in projection maintenance.
